# Drawingvoice 2.0: classroom joint designing and Facebook interactions to develop reflexivity and awareness

**DOI:** 10.1007/s11423-021-10042-3

**Published:** 2021-09-17

**Authors:** Stefania Carnevale, Immacolata Di Napoli, Francesca Esposito, Caterina Arcidiacono

**Affiliations:** 1 Department of Humanities, University of Naples Federico II, Via Porta di Massa 1, 80133 Naples, Italy; 2 Instituto de Ciências Sociais, Universidade de Lisboa, Av. Professor Aníbal de Bettencourt 9, 1600-189 Lisbon, Portugal

**Keywords:** Shared drawing, Facebook interaction, Team working, Reflexivity, Active learning approach

## Abstract

Drawingvoice 2.0 is an instructional method of collaborative pencil and paper drawing to use in the school classroom, followed by Facebook interaction on the drawing produced in class. It is based on a participatory and meta reflective approach, explicitly aimed at deconstructing, negotiating, and reconstructing the meaning that students attribute to themselves regarding their professional expectations and educational pathways. In particular, the collaborative pencil and paper drawing allows for the student’s emotional symbolisation processes underlying their educational pathway. Drawingvoice 2.0 induces a multidimensional cognitive and meta-cognitive process further supported by the following interaction on Facebook. Therefore, the World Wide Web is the added resource for sharing and deepening the classmates’ discussion. Finally, Drawingvoice 2.0 supported structural group interaction and was an important supportive and instructional method to bring about transformational and developmental training practices. As the main result, in our experience, psychology students increased their reflectivity about their strengths and threats in being psychologists within their cultural contexts and potential positive resources underlying their choice. Drawingvoice 2.0 thus enhanced their self-awareness about the lights and shadows of their training and future professional career.

## Introduction

The training career of university students is the object of many studies, and according to Eurostat (2020), there are already 40.7% of young Europeans (30–34 years) with a tertiary education qualification.

In Italy, AlmaLaurea Interuniversity Consortium Report (2019), in analysing these data, suggested that Hei (Higher Education Institutions) should wonder about effective and innovative methodologies facilitating the training processes and students’ towards the job market.

An increasing number of university courses have improved teaching by collecting, analyzing and showing "visual data" (Yoshihama & Yunomae, 2018) and visual methods using photos, drawings and paintings. Those methods allow students to express themselves in a more simple, immediate, personalized, concise and creative way. However, to improve student participation and their best performance, meta-reflective activities need to be implemented using visual and media research tools. (Kitsantas & Baylor, 2001; Nikiforos & Karakitsou, 2020; Reavey, 2012; Van Merriënboer et al., 1992).

In particular, the recent global Covid-19 pandemic has accelerated the experimentation and diffusion of innovation in teaching. This means that we face a revolution in teaching, and teaching methods should be improved to support interaction with students and their active and reflective learning (Novara et al., submitted; Marsick & Davis-Manigaulte, 2011). The educational structure must understand the needs of the students and promote their mutual interaction and trust through educational and participatory experiences (Prilleltensk, 2005).

## Related literature

### Creativity and digital tools in a training context to co-construct individual and social skills

Educational contexts share meanings and emotions that allow students to understand the “What? How? And why?" in learning and teaching processes (Meyer & Hudson, 2011).

In modern society, creativity and digital technologies have become two central resources for success and development, especially for students (Meyer & Hudson, 2011). They allow the creation of meaningful and productive interactions and open up new horizons for educational activities in the face of the social and economic changes that characterize our century (Beghetto, 2010; Craft, 2013). Furthermore, they have also become crucial educational objectives in European policies (Ferrari et al., 2009).

#### Drawing as a metacognitive creative and participatory tool in an educational context

Recent studies have shown that drawing is a significant creative activity in educational settings, and it plays an essential role in learning and communication science (Ainsworth et al., 2011; Lin et al., 2017). It visualizes affective, cognitive processing, supporting verbal explanations of complex scientific systems and processes (McCrudden & Rapp, 2017; Rau, 2017). Furthermore, learning by drawing improves metacognitive accuracy (Bobek & Tversky, 2016; Cheng & Beal, 2019; Cooper et al., 2001; Fiorella & Mayer, 2016; Leutner & Schmeck, 2014) and potentially subsequent self-regulation (Fiorella & Zhang, 2018; Schleinschok et al., 2017; Thiede et al., 2010).

Drawing, as a creative activity, regardless of its expression, graphic or digital, is considered one of the most significant constructive (Chi & Wylie, 2014) and generative learning strategies (Leutner & Schmeck, 2014; Schmeck et al., 2014; Schwamborn et al., 2010).

Drawing allows students to make sense of the information they have just acquired by selecting the most important, reorganizing it and integrating it with what was already known (Van Meter & Firetto, 2013).

In our vision, the generation of drawings, as an external visualization strategy (Lin et al., 2017), induces the integration of verbal and non-verbal representations, facilitating access to more expansive dimensions of knowledge. This is metacognition (Fiorella & Zhang, 2018); therefore, a meta-level of thought refers to a level of thought that implies active control over the thought process used in learning situations.

Furthermore, drawing during training courses allows people to "think" about activation processes, shared meanings and individual and collective stories, telling them, sharing them and "re-meaning them" (Fuks, 2010, 2016; Gillies et al., 2005).

The quality of the learners' experience is conceptualized as a complex intersection of didactic, social and cognitive presences (Szeto, 2015). But, despite the importance of all these elements for learning and metacognitive development, research programs often focus on only one approach and content.

Indeed, the metacognitive and emotional dimensions have often been investigated separately in their relationship to education, although research on affect and emotion affecting education is evident in some studies (Efklides & Volet, 2005; Pekrun et al., 2002). As Daradoumis et al. (2013) demonstrated, their joint activation has significant implications on the learning process. It produces transformative paths, both in traditional didactic contexts and in e-learning paths.

#### Drawing and reflectivity

The most recent literature (Cheng & Beal, 2019) shows that visual methods enable better learning in the learning process. Ferrer et al. (2005) have, in fact, implemented an integral transformative and experiential learning process in which all human dimensions (mind, health, spirit) are involved and where emotions, representations, expectations, stereotypes and strategies are the object of shared reflection, thanks to the design, communication and asynchronous relationship with the teacher.

In educational contexts, the Visual Voice Technique (Yonas et al., 2009, 2013) brought visual methods into schools, making them an active teaching tool which helped to explore representations and experiences of students, in order to activate them with reflective experiences to put at the service of the learning community. Indeed, visual methods support students' reflexivity and awareness of their studying and representations.

Collaborative paper and pencil drawing, proposed by Fuks (2016), is a specific tool to create shared narratives among the members of the group; the shared narratives of this group activate reflective mirroring based on the support, interpretation and connection of members' experiences, as described by Esposito et al. (2017), Esposito et al. (2018) and De Luca Picione et al. (2018). The common design activates the group mind, supporting shared creative processes. In particular, Fuks used drawing to make explicit what is implicit in relationships to build emotionally sustainable, highly formative and aware groups and paths of formation and change. This highlighted the role of generative contexts and reflective dialogue in the sharing processes (Alvensson & Skóldberg, 2000) directed by each participant in defining their own "Relational Positioning" (Fuks, 2016). Therefore, in Fuks's ecological approach, drawing becomes a tool to activate shared awareness and reflexivity. It aims to change and discover new horizons more aligned with people, their "implicit theories”, and their "shared social knowledge".

#### Social media as a supportive educational tool

Chu et al. (2019) recently found that the reflective and meta-reflective processes of students' creative learning experience have the support of Facebook as a form of communication technology; it promotes more significant interaction and collaborative learning, supporting it (Charnigo & Barnett-Ellis, 2007; Hewitt & Forte, 2006; Matthews, 2006; Mazer et al., 2007; Munoz & Towner, 2009; Ragupathi, 2011; Rego, 2009; Selwyn, 2009).

Indeed, social networks in educational settings develop peer communication, collaboration, and active learning or learning through discovery, and encourage students to co-build self-motivation (Cojocariu, 2012; McCombs & Whistler, 1997; O’Neill & McMahon, 2005).

Certainly, the Web 2.0 revolution, which permeates people's daily lives, making information and knowledge increasingly shareable and immediate (Procentese et al., 2019a, b, c) and the current culture that is increasingly visual, has also entered the world of education. Indeed, it supports the idea that users add value through their participation (Mason & Rennie, 2008) and facilitate both individual and socially mediated metacognitive and reflective practices (Biasutti & Frate, 2018; Layen & Hattingh, 2018; Lin et al., 1999; Snyder, 2019).

### Drawingvoice 2.0: research aims and questions

Drawingvoice 2.0, as a teaching method, allows students to activate metacognitive emotional thinking in their training process, making them more aware and responsible, using creative visual expressions also supported by modern communication technologies. The method is characterized by an ad hoc format in which group activities, collaborative pencil and paper drawing (Cigoli, 2018; Fuks, 2009) and asynchronous communication are combined.

Drawingvoice 2.0 consists of a variable number of group sessions for the class and conducted by a teacher in the role of facilitator. It aims to encourage students to share their feelings and thoughts using rational, imaginary and media tools to induce a transition from rational to emotional thinking and from the verbal to the non-verbal channel.

This methodological format provides the opportunity to explore and share emotions, representations, expectations, stereotypes and learning strategies, and helps to co-build more aware, authentic and therefore more effective training with students.

Consistent with the fruitfulness of sharing emotional and cognitive representations among people proposed by Fuks (2009), we have structured this method of active learning and participatory teaching by combining creative and expressive skills in class work and consequent social web interactions. We have introduced participatory web action because sometimes the classrooms are overcrowded, or the students don't have time or are not in the mood to activate their emotional thoughts. Therefore, adding a specific moment of virtual interaction for the whole group of students means proposing a strengthening tool for class interaction. At the same time, it proposes a teaching strategy that is also useful for distance learning, overcoming the lack of interaction between peers that online teaching sometimes entails.

Drawingvoice 2.0 is part of the teamwork facilitation method developed by Saúl Ignazio Fuks (2009, 2016), and its peculiarity is to have introduced web support in the "Group facilitation methodologies for the promotion of reflective contexts" by Fuks (Arcidiacono, 2017; Fuks, 2010).

Synchronous and asynchronous modes in teaching have demonstrated the effectiveness of training experiences by sharing spaces that go beyond physical boundaries and the limited time of face-to-face teaching (Hrastinski, 2008; Moore et al., 2011; Tavangarian et al., 2004; Welsh et al., 2003; Zhang et al., 2004).

In this case, it improves the reflexivity process by allowing the opportunity to rethink the didactic content in a shared approach that, thanks to social media, further connects all class members. Its final purpose is to activate the awareness that the rational can reach the emotional domain allowing group discussion and comparing individual thoughts and feelings. Therefore, collaborative drawing is a tool for participatory growth and learning by sharing emotional experiences.

This article aims to describe the Drawingvoice teaching method and explain how drawing as a visual tool and online interaction as a participatory tool meet different spheres of knowledge through emotional experiences and unveil resources to facilitate a more solid and sustainable training course.

The research question was:

- Do small class group drawing sessions and online class communication support teaching and the co-building of knowledge and sharing emotional experiences? More specifically, is collaborative drawing in small groups functional for students to focus on their resources and obstacles? And finally, is the additional interaction on Facebook able to promote more participatory and reflective thoughts? More specifically, is the small group collaborative drawing helpful for students in focusing their resources and obstacles? And finally, is the Facebook interaction able to promote more participatory and reflective thoughts?

## Method

To answer these questions and evaluate a new use of online classroom connections in an integrated procedure of classroom work and sharing on the web, specific action research was carried out (Arcidiacono et al., 2016a, b).

Two exercises took place, each one on a specific topic, but both interrelated and characterized by discussion and joint design in small groups and a subsequent discussion in a larger multimedia group.

The objects of reflection and representation of the exercises were:

- *In Exercise I*: the representation students had of themselves as psychologists: their fears and hopes;
- *In Exercise II*: the professional tools they wish to take with them in their professional future. Each activity lasted about 3 h.

### Procedures

The activities were structured in a three-step process in which students were involved in working together in the classroom and on the website. During these steps, a shift from rational thinking to the emotional and verbal channel to the non-verbal one was promoted to give voice to the various dimensions (rational, emotional, representational, perceptional, etc.).

**Fig. 1:**
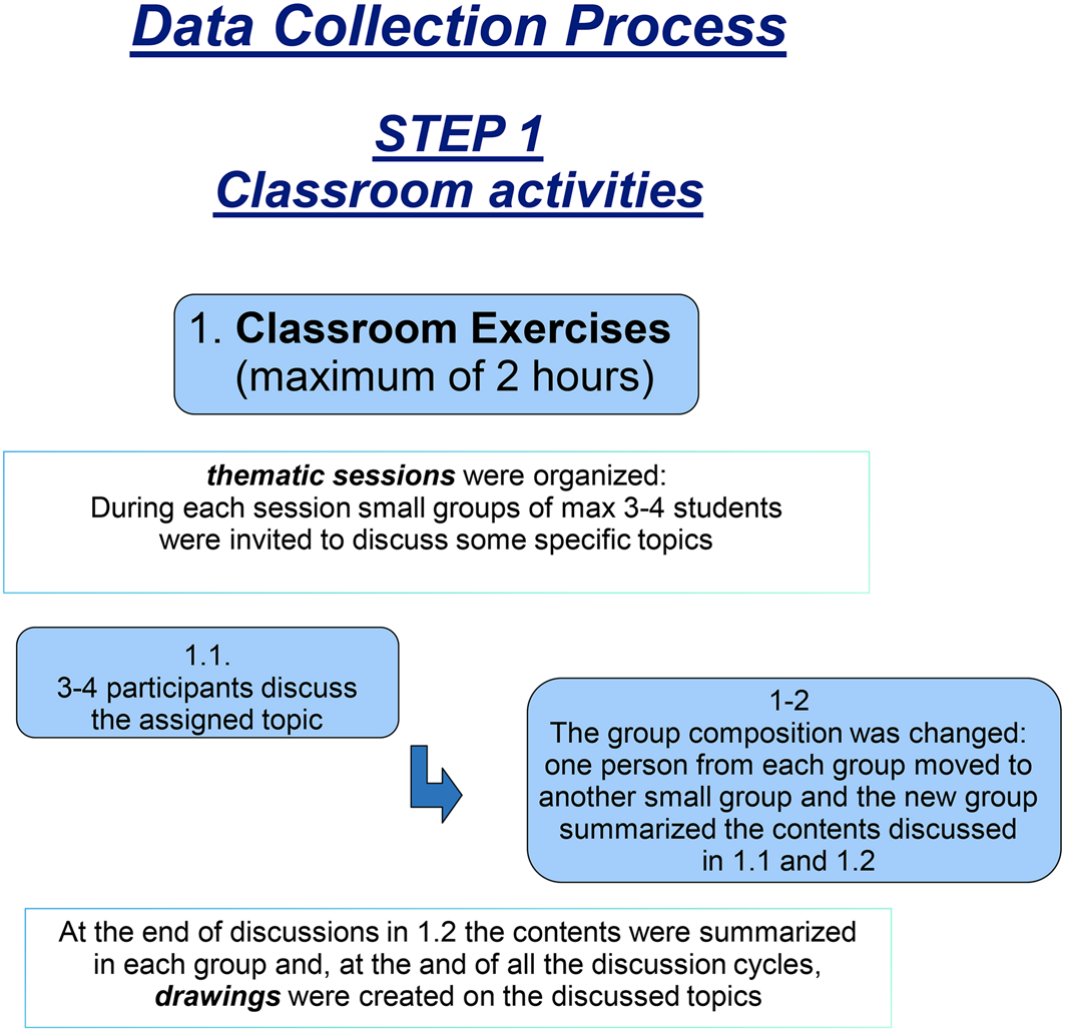
This figure illustrates all the classroom activities during phase 1

**Fig. 2:**
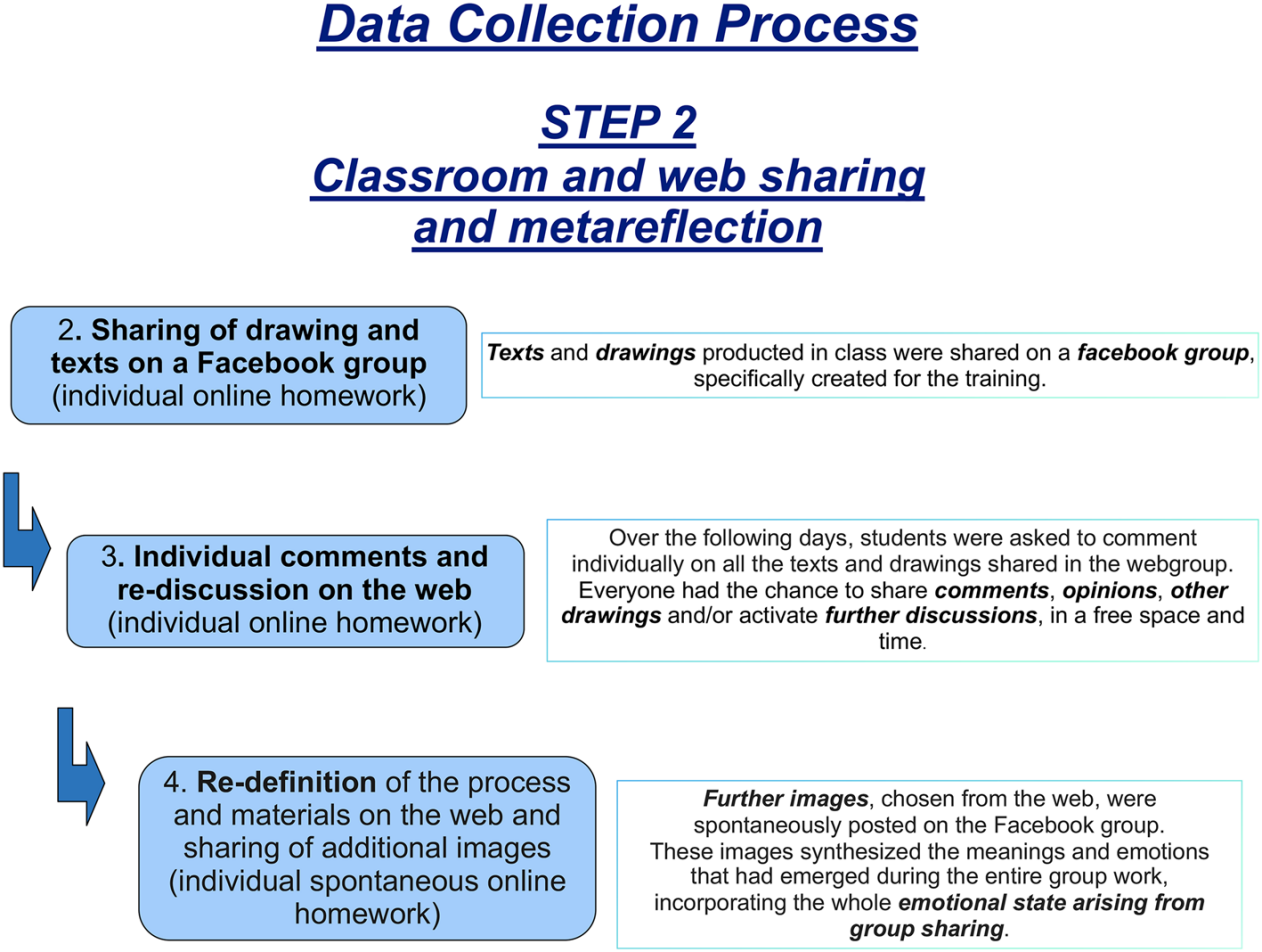
This figure illustrates all the meta reflective classroom and web sharing activities

This process was divided into various activities further described in the following paragraphs:

*Phase 1—classroom activities* (see Fig. 1). The classroom training (lasting a maximum of 2 h total) consisted of a discussion in small groups (max 3–6 participants), followed by a small joint drawing session on the specific topic. This procedure consisted of five subsections or steps:

*Phase 2—Classroom and web sharing as well as meta-reflection* (see Fig. 2). In this phase, a Facebook account with the same name as the course was created specifically for the training to share all the materials produced during Phase 1.

The goal was to activate a space for re-elaboration, examination, sharing, comparison and co-construction of knowledge, which would expand areas and times of interaction beyond the two lessons in the classroom.

**Fig. 3:**
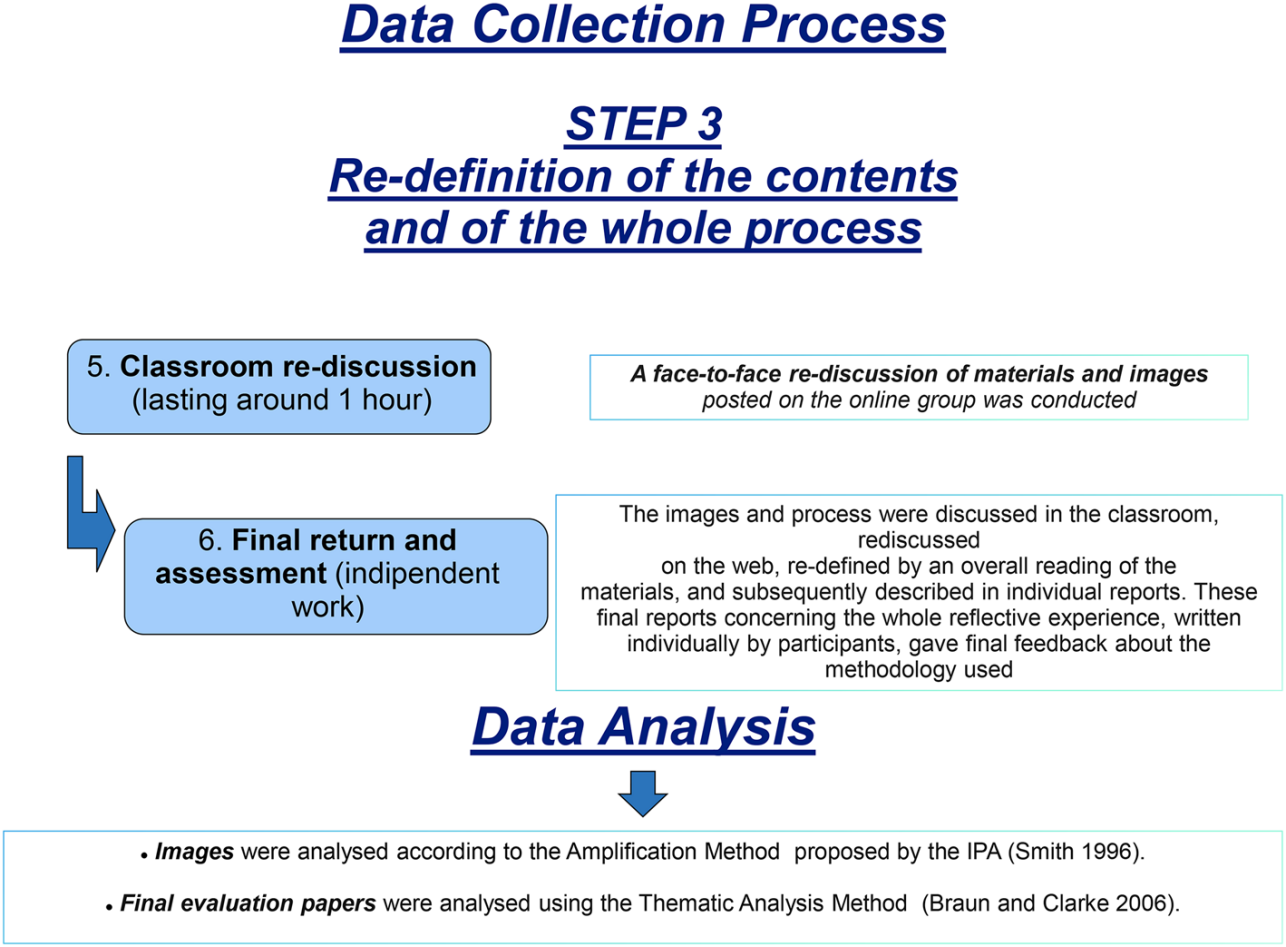
This figure illustrates the re-definition activities of the contents and the whole process

*Phase 3—Re-definition of the contents and the whole process* (see Fig. 3). A face-to-face *classroom re-discussion* took place (lasting about 1 h) on the materials and images included in the online group. The entire process also became the subject of discussion. Furthermore, in a *final evaluation*, many students discussed the results, and their reactions and suggestions were collected and analyzed.

During Exercise I, the groups of students produced 12 drawings, while during Exercise II, there were 8. An image for each exercise was also uploaded spontaneously by the individual participants at the end of the online discussion procedure.

### Participants

Forty-nine students participated in this shared reflexivity experience; they were mainly females (84%) aged between 22 and 50 (average 23, median 25). They attended the ‘Methodology and Techniques of Community Psychology’ module of the Master's degree course in Dynamic Clinical and Community Psychology at the University of Naples—Federico II. The length of the whole module was 3 months, with attendance of three sessions a week.

The group of participants represents the gender distribution among students enrolled in the degree course in Psychology. According to the qualitative approach (Charmaz, 2006), the aim was not the representativeness but the exploration of the students’ world of meaning and the transformative value of an innovative methodology.

### Data analysis

Each session conducted by the class teacher was supported from time to time by a specific student in the role of observer, who took notes on the explanations of the drawings made by the authors in the classroom, writing a detailed report at the end of each session.

These texts were then used to analyze the drawings and the process itself to understand their shared contents and meanings.

The drawings were finally analyzed by the teacher and by all the groups of students according to the Interpretative Phenomenological Analysis (Smith, 1996); the following phases of analysis were:

1. *Reading of texts, reports and drawings* produced to familiarize themselves with the data and produce an initial detailed view of the content addressed and shared information. This phase was essential to make the individual group the focus of the analysis and become familiar with its narratives, both written and drawn.
2. *Multi-level semantic content analysis (amplification*). Three types of annotations were produced: descriptive, linguistic and conceptual comments.
3. *Identification of emerging issues*

Relevant dimensions and issues were identified about the theme of the year. They were organized into tables that allowed them to be grouped into macro-categories. Therefore, this analysis process was both analytical and theoretical, as it allowed us to interpret both textual and non-textual material, creating connections between emerging themes and to co-construct further knowledge.

The evaluation papers presented by the students at the end of these activities were analyzed by the whole research team using the Thematic Analysis Method (Braun & Clarke, 2006), following a progressive process structured in 6 phases: Familiarization with the data; Generation of initial codes; Search for themes; Evaluation of internal homogeneity and external heterogeneity; Definition of themes and sub-themes; Drafting of reports.

## Results

We carried out the elaboration and analysis of materials with the procedure indicated in the previous paragraph, and for each exercise, the results were illustrated as following:

*Exercise I* Becoming psychologists: motivation, hopes and fears of psychology students.

**Fig. 4:**
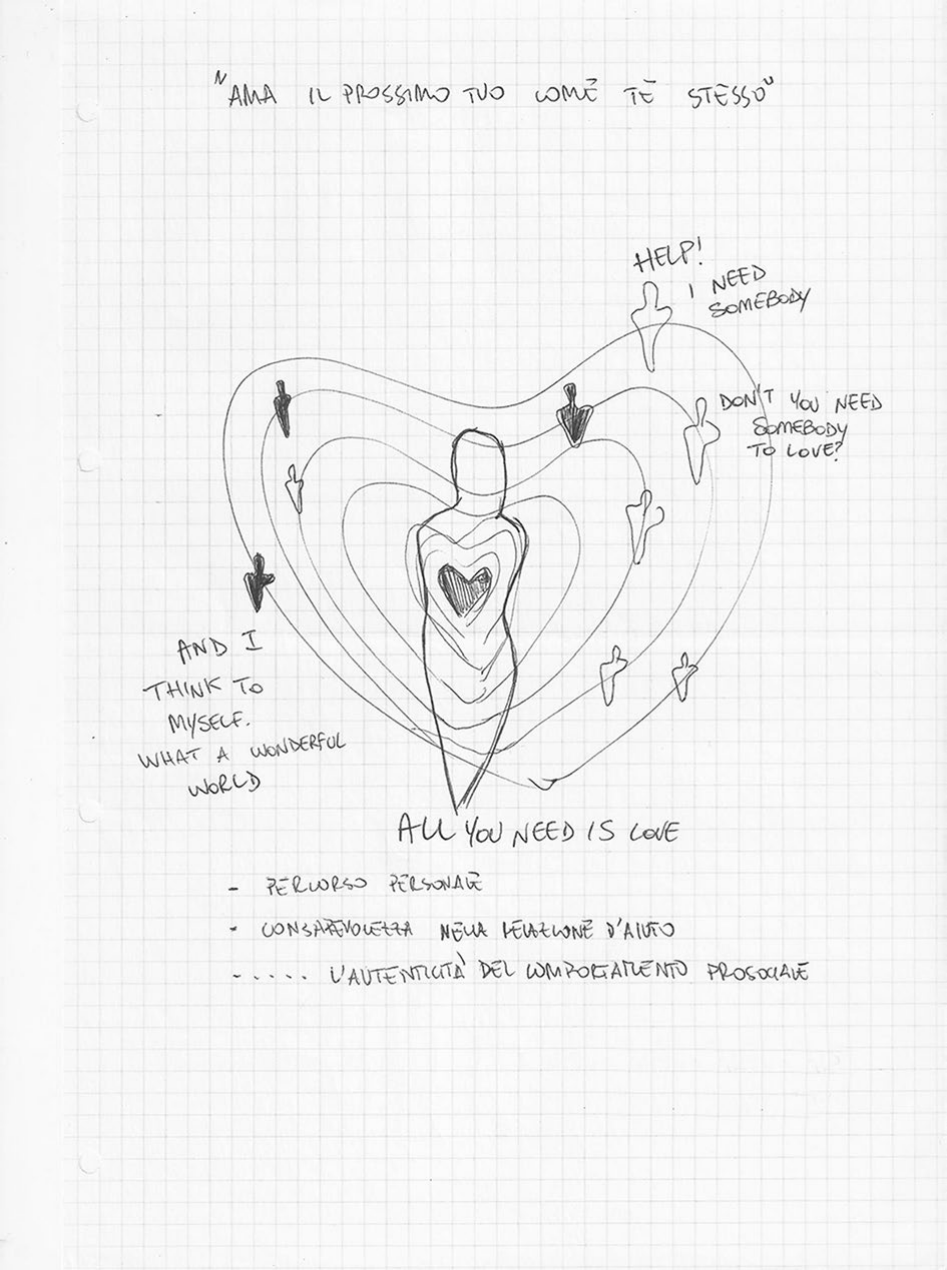
This image portrays a body whose heart branches out towards the outside in a system of relationships that take on the juvenile register of music. Then there are lines from famous love songs quoted in English: "*Help*", "*I need somebody*", "*Don’t you need somebody to love?*", and “*All you need is love*"

**Fig. 5:**
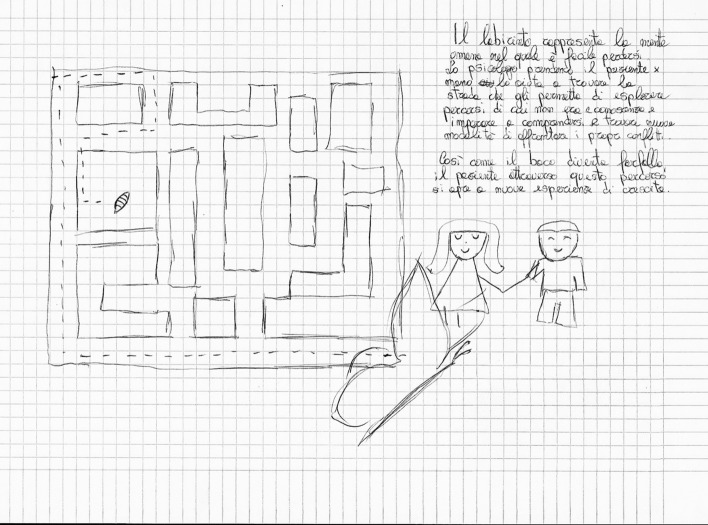
In this drawing **“**The labyrinth of change” is represented. This image depicts Psychology as a labyrinth with transformative and growth power: in fact, it is crossed by a caterpillar which, in the end, emerges as a butterfly

**Fig. 6:**
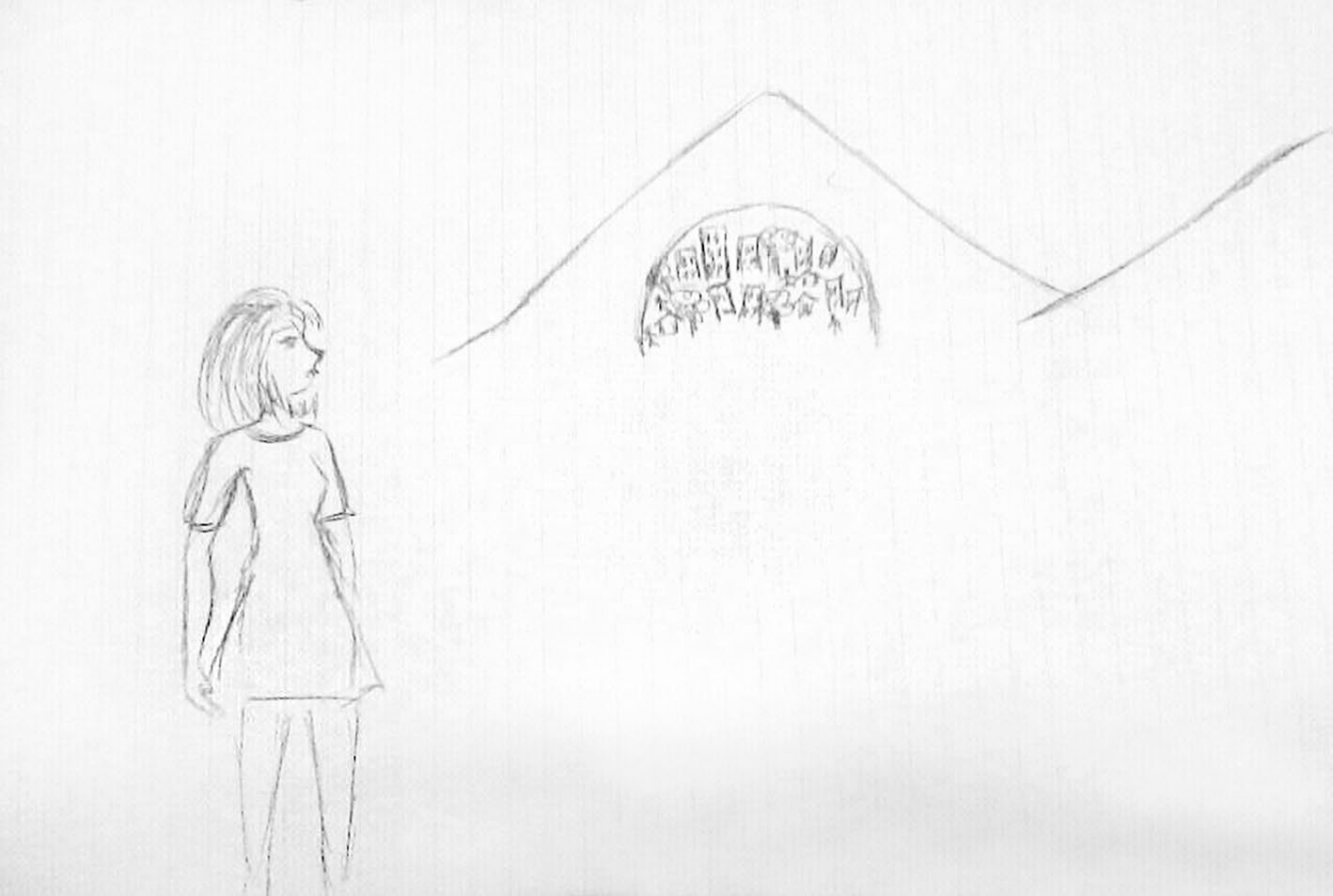
This drawing was called “The gap that allows to look beyond” and it portrays a woman looking towards a mountain behind her

**Fig. 7:**
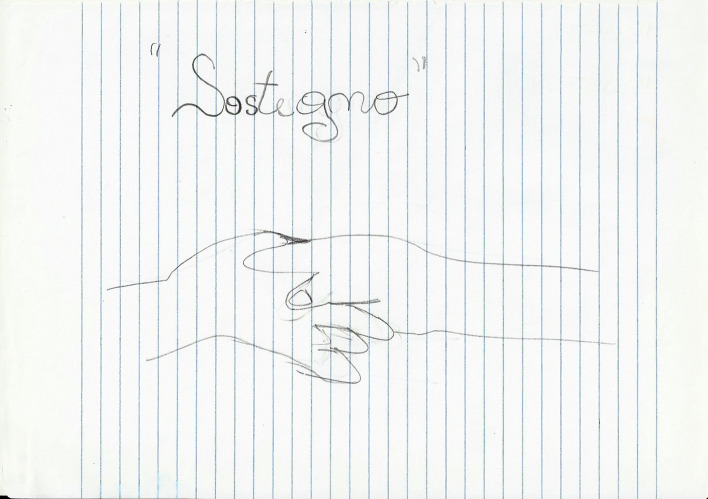
This drawing depicts “Support” and shows two hands that meet and hold each other

**Fig. 8:**
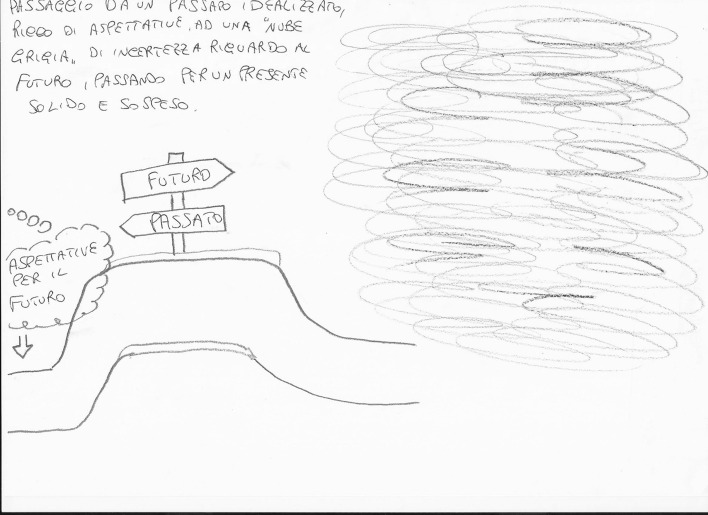
This drawing was named “A bridge toward a cloudy future”; in fact, the image illustrates a solid bridge on which there are arrows in two directions: one towards the past and the other towards the future. On the side of the past, the "*Future expectations*" are imagined, in a cartoon, while a grey cloud is drawn on the side of the future

In *Exercise I,* the drawings focused on four main themes:

1. *The students’ personal feelings* about their emotional world. On the whole, these drawings expressed the students’ goals (Fig. 4) and, at the same time, their undefined fears about their training path (Fig. 5).
2. *The Psychologist* (Fig. 6) The Psychologist (Fig. 6) was represented through their competence in “*looking beyond*”, thanks to the knowledge of the psychological dynamics that underlie many aspects of inner life. Their main functions were to guide and support them in entering the labyrinth of the psyche to discover the “latent” part of the processes of growth and change. They were therefore designed as a relationship expert at various systemic levels and as a professional able to reveal the unspoken aspects of the context.
3. *The clinical process* seemed to be a labyrinth in which it was easy to get lost. Still, with the help of the psychologist, it was possible to acknowledge its transformative potential. The main element of this process was the competence of the professional (Fig. 7) that allowed access to revealing the “dark part of the Self”.
4. *The importance of professional training* was caught in its “transition" to professional life (Fig. 8). It was drawn as a safe bridge supported by the bricks of the university, experienced by students as a maternal element that protects and sustains. Its solidity seemed to clash with a cloud of uncertainty and mistrust that reveal the impossibility of seeing into the future, understood as the lack of hope in realizing all the expectations that have supported the choice of the training path.

*Exercise II* Basic psychological competencies.

**Fig. 9:**
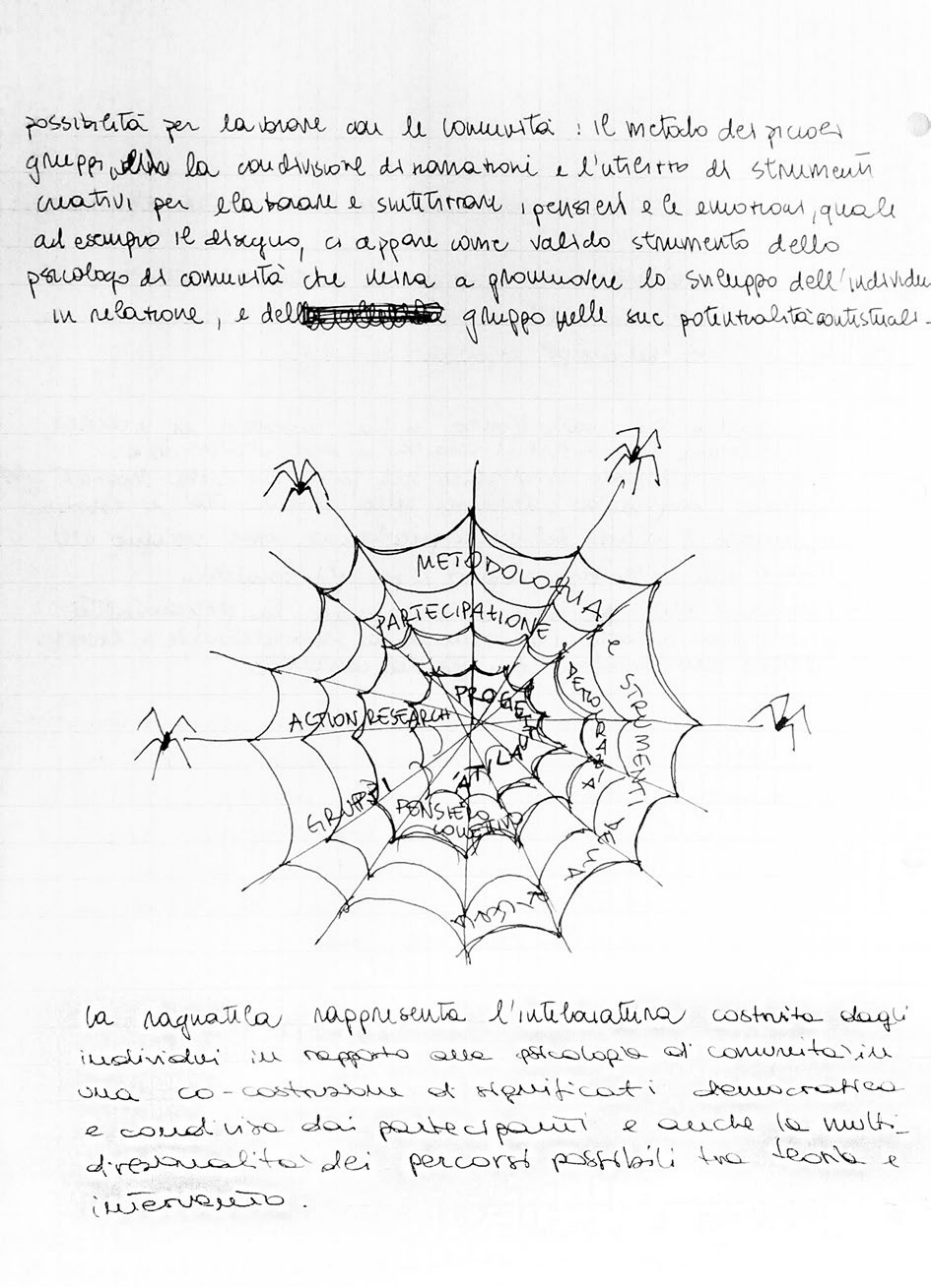
“The spider web of interaction” is the title given to this drawing; it represents the spider web symbolizing the "*framework, made up of individuals and their interrelation*s”. Within the interweaving of the spider web, the methodologies and tools of Community Psychology, such as *Action-Research, Methodology and Research Tools, Participation and Democracy, Collective thinking, Groups, and Projects* are depicted

**Fig. 10:**
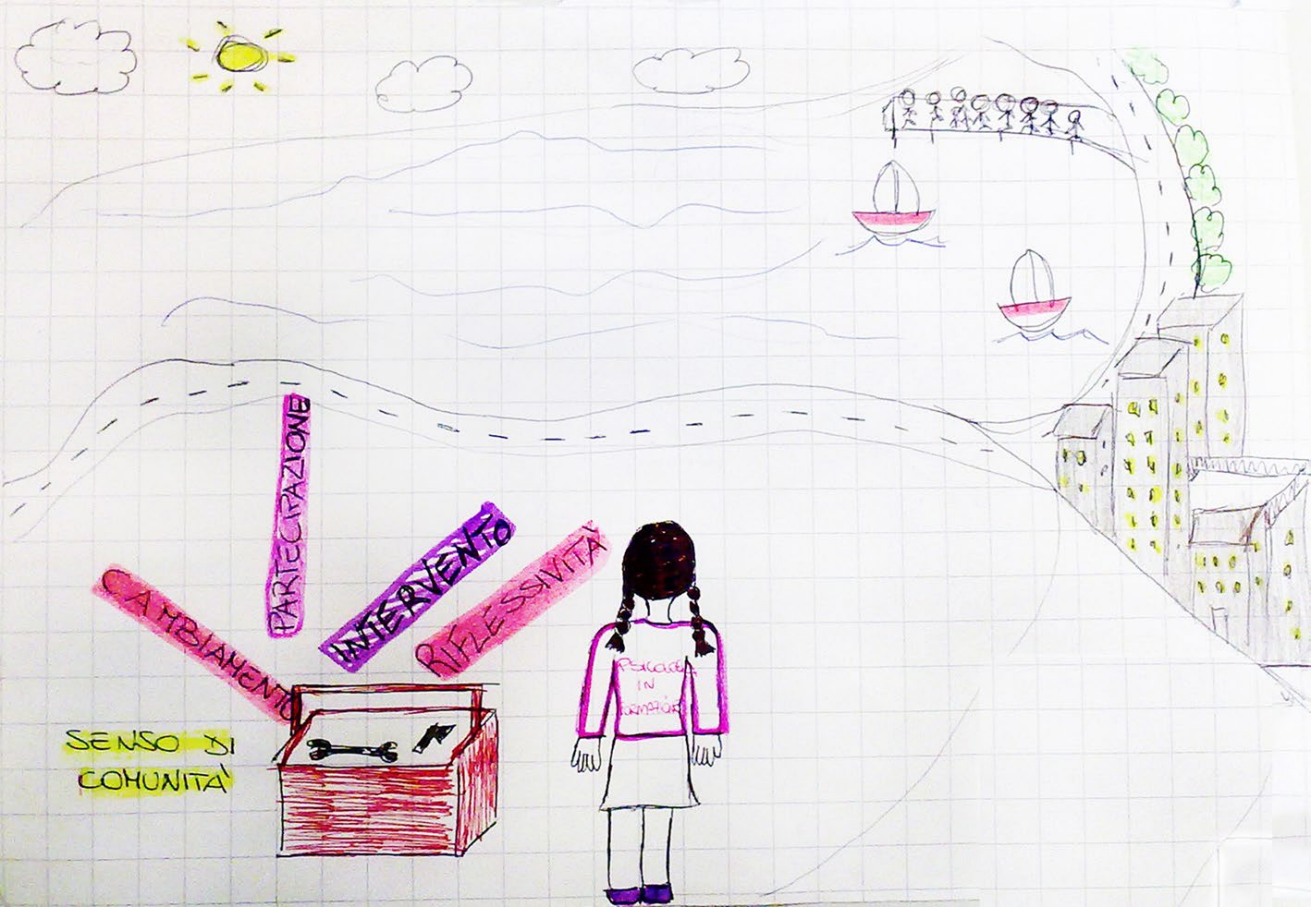
This image depicts “The Psychologist’s toolbox” (containing reflexivity, intervention, participation, change, sense of community) that designs the professional future. The figure represents the figure of a psychologist in training looking towards a landscape made of sea, sun, buildings and streets. Next to him is a "box" of instruments that take the name of the identification elements of the Action Research selected by the group. These are *Sense of Community, Change, Participation, Intervention, Reflection*

This exercise highlighted two points:

- *The process of action research*, represented by a circle or group of people, emphasized the democratic, participatory and inclusive approach of Action Research in community psychology (Arcidiacono et al., 2016a, b). The diversity of perspectives and the opportunity to "see with different eyes" were highlighted through the awareness and responsible process of sharing, negotiating and confronting the internal and external relational dynamics.
  This process was captured in its *connective dimension* thanks to the metaphor of the spider's web (Fig. 9), which expresses the interdependence among the activated dynamics, and marks exchange and discussion to activate change, regeneration and empowerment.
- The *psychologist* was represented in their educational and professional dimension; in this way, training became a clear landscape that opens up in front of the psychologist. Students find themselves reflecting in a meta-reflexive dynamic, bringing with them the tools learned through study. The Psychologist’s “toolbox” (containing reflexivity, intervention, participation, change, sense of community) designs the professional future (Fig. 10).

**Fig. 11:**
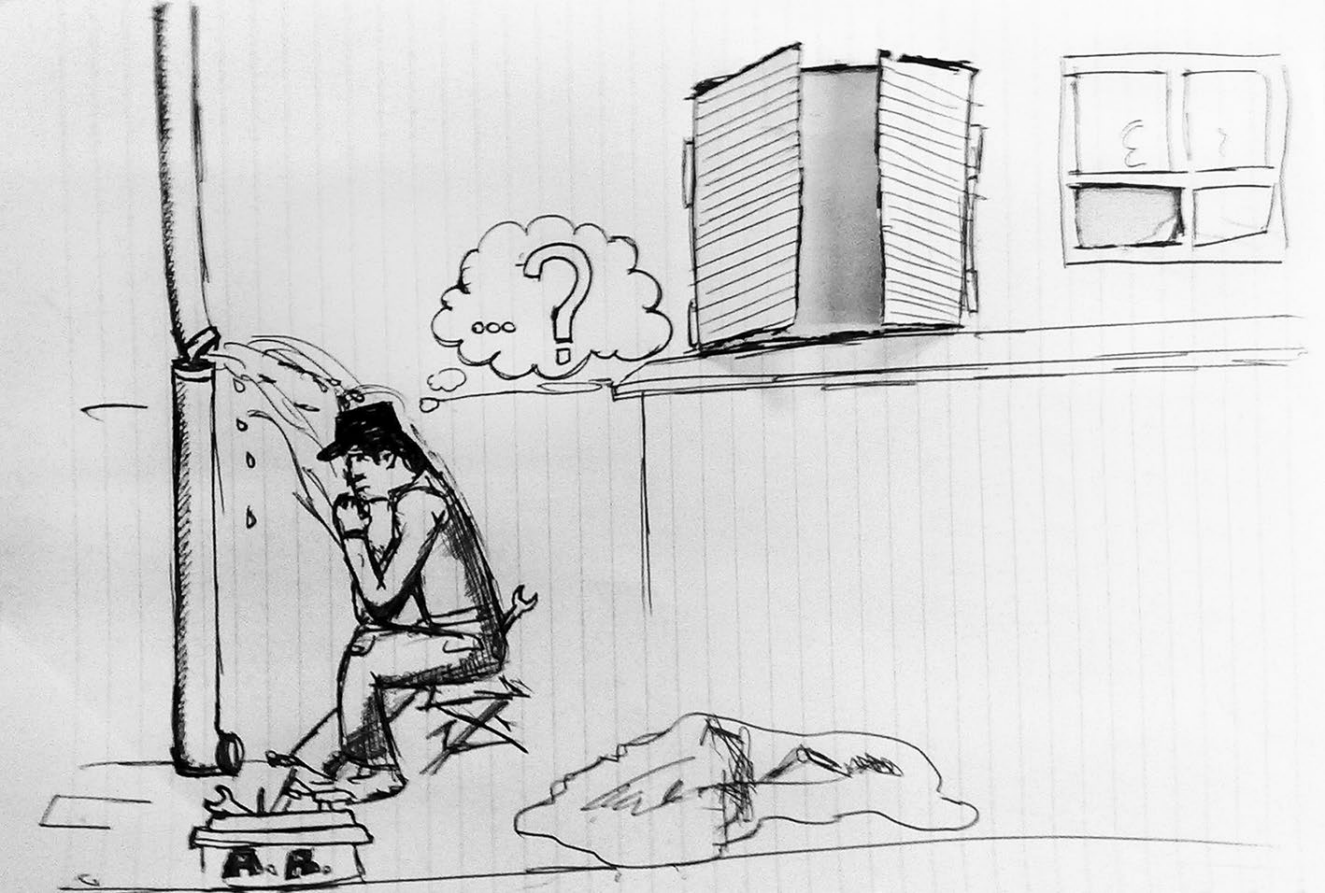
This drawing was titled “The Reflective Plumber”. It represents a plumber in front of a broken pipe; while the water gushes onto him, he reflects on how and with what to intervene, having beside him his box of work tools. In the background, a window is drawn. It represents the "third eye" that the psychologist brings to contexts, taking part in them

Moreover, with the metaphor of the plumber (Fig. 11), the professional was defined as an expert who, participating in the context, brought a "third eye" reflecting with people.

The drawings showed “*know-how*" not delivered from the top by a de-contextualized expert but co-constructed with people via the skills of researcher-psychologists, stimulating transformative regeneration processes for the common good.

## Discussion

The aim was to activate an aware and conscious learning process by asking students to express their vision of the discussed topic through drawing. At the same time, psychological knowledge and know-how were co-constructed and shared in face-to-face activities and thanks to subsequent Facebook interactions.

This allowed students to share their feelings, thoughts, and cognition with their future role as psychologists and allowed them to participate in a participatory, multidimensional, multimedia and interactive teaching path, co-building it together.

In this way, psychological knowledge and know-how were co-constructed and shared in face-to-face activities and web experiences.

The ultimate goal was to create exchanges and comparisons with transformative value, promoting reflection and awareness in research and training, based on dialogue and the co-construction of knowledge, competence and emotional knowledge. According to Chu et al. (2019), the reflective and meta-reflective processes of the students’ creative learning experience with the support of communication technology promote more significant interaction and collaborative learning while also supporting it. Moreover, these tools prove the effectiveness of training experiences. (Francescato et al., 2006; Hrastinski, 2008; Moore et al., 2011; Tavangarian et al., 2004; Welsh et al., 2003; Zhang et al., 2004).

The three most significant conceptual themes which emerged by analyzing the final reports (Thematic Analysis of student’s report) written by the students, related to the educational and learning path experienced during the Course. The conceptual themes were the following:

- *The use of drawing as a tool for expression* The students described how their thoughts on experience, the potential of drawing and creativity in an active and participatory way changed day by day along with the new instrument.
- *The Web as a parallel supporting space* Creating a media group to continue discussing the drawings and sharing this experience with others was initially accepted with some doubts.

The students had always seen Facebook as a space for recreation and informal communication between friends. At the beginning of the course, all the students underlined the risks and the criticalities regarding Facebook’s positive aspects and potential. But in the end, the social network became a real resource:

Facebook was our means of communication, the representative of a collective work. […] It contributed to keeping the group always in contact; it allowed us to share information, videos, links, articles on classroom issues; it fostered comparison and dialogue through the exchange of comments and opinions (student, female, 24 years old).

Using the words of Mantovani (2004), the web support allowed the participants to "*build a new 'we', more solid, more informed, more vital*", but also more participatory and empowered in its internal resources, strengthened precisely by awareness and the strength of collective thinking.

Web support has become precious space–time continuity, capable of guaranteeing relational continuity and an open circuit of knowledge.

- *An innovative and engaging community psychology course*. The most significant and representative elements of the experience being empowering and exciting were innovation, participation and reflection.

*Sharing, comparison and transformation* were the words that dominated all the texts. *Teamwork* was recognized as the common thread of the whole process, establishing itself as a model of critical, multi-perspective and co-constructive interaction founded on awareness, reflexivity and negotiation.

**Fig. 12:**
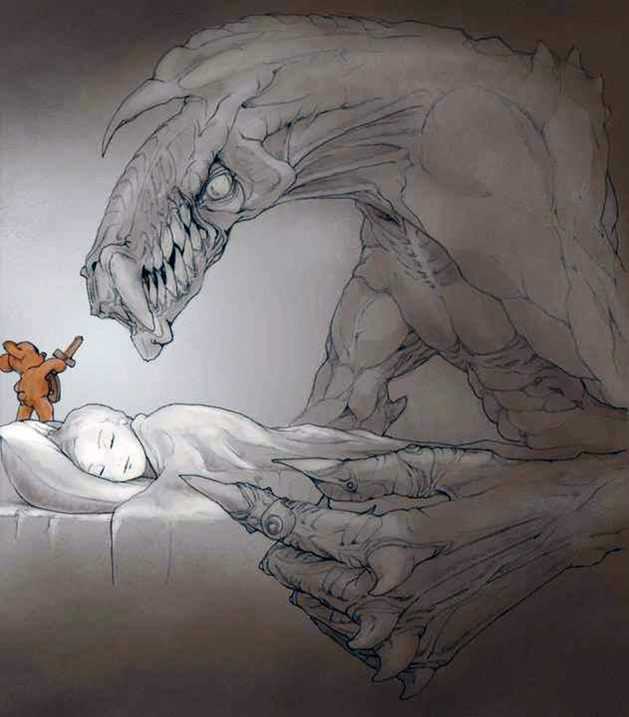
This image represents “The Guardian of dreams”. It portrays a little girl sleeping in her bed and who, on one side, is besieged by a horrendous monster. On the other side, she is defended by a tiny teddy bear wielding its sword against the fearsome adversary, fearless and unafraid of its intensity

**Fig. 13:**
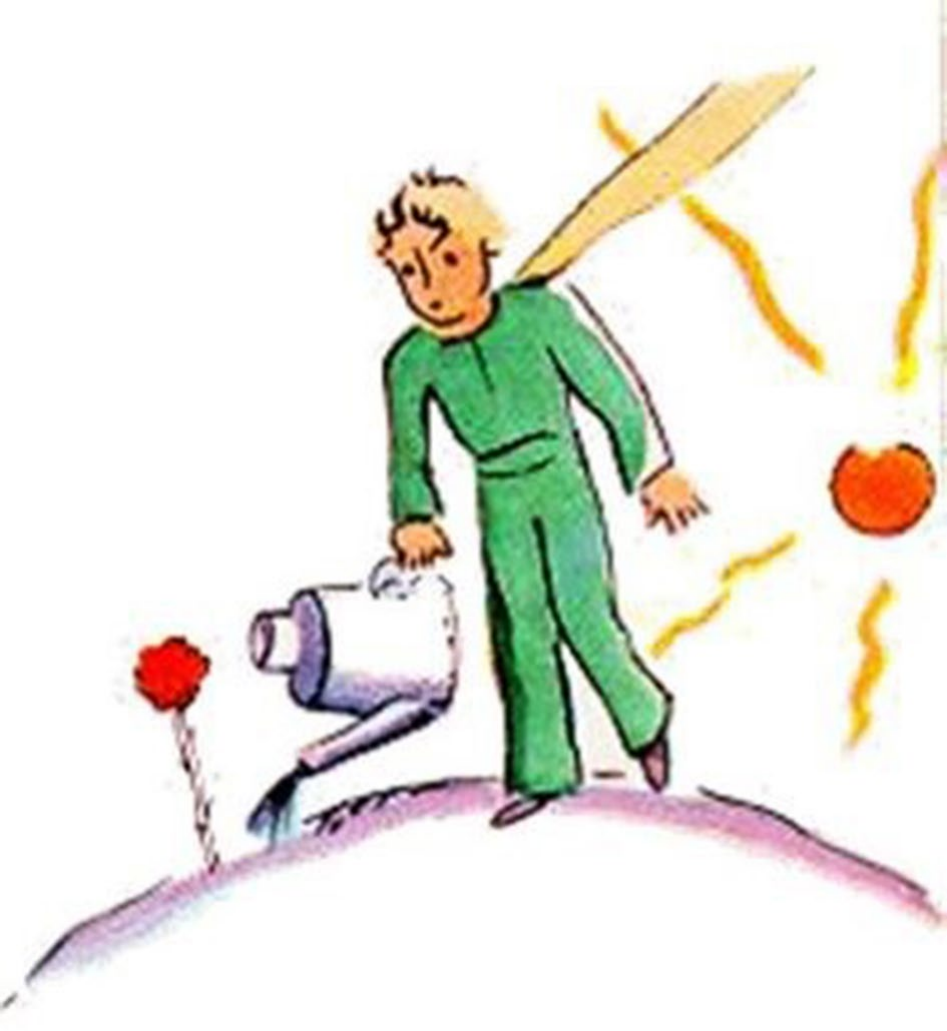
This figure depicts “The Little Prince watering his rose”. It hails from "*Le Petit Prince*" (Antoine de Saint-Exupéry, 1943) and illustrates the Little Prince, the protagonist, watering and taking care of his rose

The results highlighted a transformative path from the drawings: (a) students moved from a focus on the negative as shown in Fig. 8 (the psychologist projected towards an undefined future covered by a cloud of uncertainty and impotence,) to the awareness of their future that became much clearer and not such a hostile landscape (see Fig. 10); (b) They *represented their role* as psychologists, from a vision centered around the emotional, empathic and support skills of the psychologist (Fig. 4) to the representation of the psychologist/researcher as a professional with skills, tools and techniques (see Fig. 11). (c) Finally, *they re-signified all the processes*: Fig. 12 showed a small teddy bear standing as ‘the guardian of dreams’, as a defender of the students’ emotional world, and an image of the Little Prince watering his rose expressed the awareness that it was time to nurture, and the moment to enhance one’s passions and emotions, which made their training a solid experience of self-development and personal and cultural growth (Fig. 13).

The collaborative drawing and the subsequent online interaction became a teaching tool to co-construct knowledge, share emotional experiences and help students in focusing their resources and obstacles, building more lasting knowledge, as testified by the students themselves during the final briefings and through the reports written and delivered at the end of the course.

**Fig. 14:**
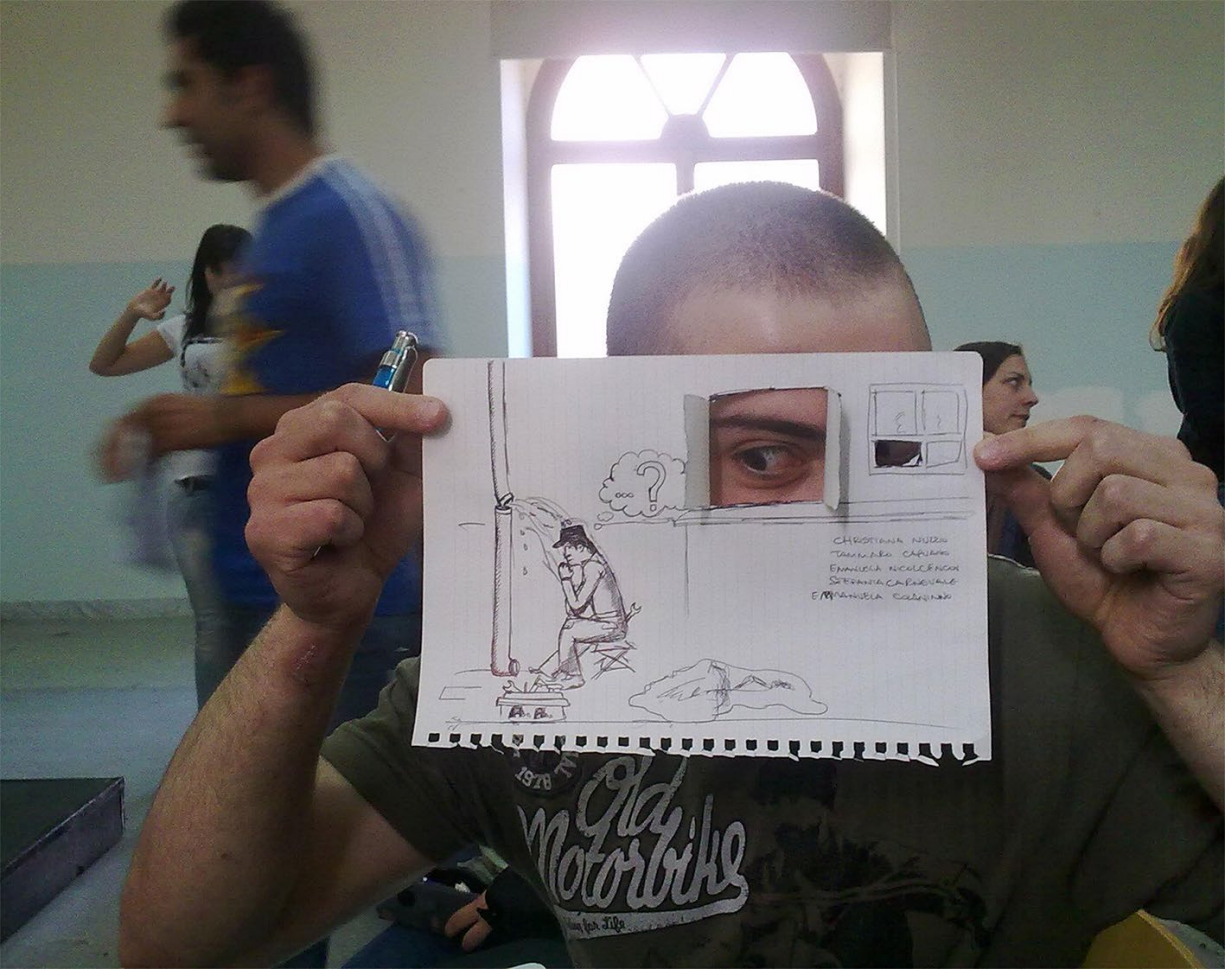
This photo illustrates “The trainee as reflective Plumber”, in which a student showed how the drawing had given them the opportunity to express themselves also through the three-dimensionality of the sheet of paper, which became, in the hands of the students, a reasonable means of communication at all levels

Indeed, the transformative value of Drawingvoice 2.0 consists of activating in the students an increased awareness and acquisition of new meaning about their training-professional path and their future perspectives. Here it was significant to add a drawing made on the last day of the course by one of the students (Fig. 14). It summarizes the whole process of introducing a single student to the training procedure.

Finally, the Drawingvoice 2.0 method is considered to be an experience of "cooperative learning" in which the teacher is not merely a transmitter of content. Moreover, learning-teaching relationships are never simple interactions but emotional-relational exchanges, constructions of meanings. According to a transdisciplinary and networking approach, learning is supported by multiple teaching intertwining in a "plural" way of mind and body, experience and reflexivity.

### Limitations

The generalizability of the study results is subject to limitations because it is a qualitative and situated study; the student participants do not fully represent the views and insights applicable to other settings and parts of the world. The group of participants was not gender-balanced, and the type of sampling was not probabilistic.

The results are correlated to specific geographic locations and specific university contexts and fields.

The validity of the methodology was inferred from complex indicators, such as the average high level of exam marks (28/30); the number of students who participated in the first exam session (95%); the analysis of the drawings that highlighted its transformative value. Furthermore, the soft indicators considered were: the analysis of student reports that may have been "filtered" by emotional participation in the experience and enthusiasm for a new way of teaching. Despite this, further applications are required to confirm the validity and transformative value of the Drawingvoice 2.0 methodology.

## Conclusions

We referred to Drawingvoice 2.0 as a teaching method within an interactive, participatory and reflective methodology acquired by Fuks (2009). The combined use of the joint drawing class activity with web interaction and face-to-face dialogue in small class groups was innovative; it pursued a conscientisation aimed at responsible and conscious change and individual and collective well-being (Di Martino et al., 2018).

Drawingvoice 2.0, thanks to the Facebook group created relationships of continuity and sharing among students. The potential of using web 2.0 in educational paths is already known in literature, and even if a solid educational value characterizes them, they are underestimated (Curtis & Lawson, 2001; Finn et al., 2019; Song et al., 2004).

The group discussion of drawing materials allowed new meanings on both subjective and group levels (Galli et al., 2017, 2018; Tuselli et al., 2015). Asynchronous thought shared online is the unique feature of the Drawingvoice 2.0 method that powerfully strengthened the sharing of face-to-face group interaction.

Therefore, the results show that.

1. Visual methods and, in particular, their combined use with online class communication support teaching by co-constructing knowledge and sharing emotional experiences.
2. The visual teaching method is beneficial for students in focusing their resources and obstacles.
3. Facebook support allows the promotion of more participatory and reflective interactions among students and between students and teachers.

Drawingvoice 2.0 offered the opportunity to activate transformative practices with a critical approach and made communication qualitatively more immediate, fluid, understandable and full of content. It allowed the co-construction of a meta-level of thought that activated collective change processes capable of promoting common well-being, relational skills and emotional awareness.

It is to be mentioned that the drawing activity in class and the following discussion on Facebook was supported by:

- presenting the methodology clearly and explicitly with times, methods, and activities to be done together, activating involvement and curiosity;
- stimulating the production, reflection and exchange of ideas, emotions and emerging representations through the management of small comparison groups to be changed in various steps.
- creating a climate of trust and equality in promoting the sharing of ideas, knowledge, emotions, experiences, feelings, affections, feelings, moods.
- encouraging group work, facilitating emotional exchange at an individual and collective level;
- at the moment of drawing, some materials were provided (sheets, posters, colours, pencils, pens) to enable the students to express themselves as freely as possible;
- the sharing and the discussions on the Facebook group by actively participating was supported;
- activating a shared space of thought, at multiple levels, on the texts and drawings produced, and a meta-reflection on all the phases.

This methodology, through the joint use of these combined procedures, permits the intervention at two levels of the training process:

1. It enables students to take on "other" perspectives to co-construct a conscious and responsible learning process at multiple levels (Arcidiacono, 2017; Dwyer et al., 2017; Fine, 2015; Kim et al., 2019; Procentese et al., 2019a, b, c; Procentese & Gatti, 2019; Van Merriënboer et al., 1992).
2. It allows the activation of a process of conscientisation through metacognition and meta-reflection experiences supported by the online sharing and ultimate further reflection that activated emotional thoughts on the learning path.

Therefore, we will propose Drawingvoice 2.0 as an awareness-raising method capable of recognizing and managing implicit knowledge and improving self-awareness about professional decision-making. The final goal is to offer this procedure as an instrument in decision-making processes for the professional dealing with quick, emotional, stereotypical, unconscious and slow, effortful, logical, conscious thoughts, as described by Kahneman (2011), as well as on unconscious symbolic meaning attribution (Mannarini & Salvatore, 2019).

The use of the Drawingvoice method acquires new importance by the wide diffusion of online teaching. From this use of Drawingvoice 2.0, we observed that education is more powerful when facilitating group interaction and discussion, as shown by Novara et al. (submitted). Online collaborative learning is a challenge in distance learning.

Therefore, a teaching strategy that includes class small group interaction and more comprehensive online interaction and reflection may empower the learning procedure. Moreover, in distance learning, the shared drawing activity may be done by small online subgroups if the teaching platform allows it. The strong message of our proposal is to implement teaching reflective participatory opportunities combining cognitive and emotional knowledge and participatory opportunity.

For future applications, we emphasize that drawing, teamwork and web interaction are the keys to this teaching method applicable to all training contexts by teachers supported by psychological and group interaction knowledge.

Moreover, in online teaching, Facebook as a shared platform will allow intergroup communication. At the same time, the classroom small team activity may be proposed for online use. In this way, reflectivity will, also be enhanced in distance learning, where pair/group interaction is usually reduced.
